# Safety, Tolerability, and Population Pharmacokinetics of Intravenous and Oral Isavuconazonium Sulfate in Pediatric Patients

**DOI:** 10.1128/AAC.00290-21

**Published:** 2021-07-16

**Authors:** Antonio C. Arrieta, Michael Neely, J. Christopher Day, Susan R. Rheingold, Paul K. Sue, William J. Muller, Lara A. Danziger-Isakov, Julie Chu, Inci Yildirim, Grace A. McComsey, Haydar A. Frangoul, Tempe K. Chen, Victoria A. Statler, William J. Steinbach, Dwight E. Yin, Kamal Hamed, Mark E. Jones, Christopher Lademacher, Amit Desai, Kelley Micklus, Desiree Leiva Phillips, Laura L. Kovanda, Thomas J. Walsh

**Affiliations:** a Children’s Hospital of Orange County, Orange, California, USA; b Children’s Hospital Los Angeles, University of Southern California, Los Angeles, California, USA; c Children’s Mercy Kansas City, University of Missouri-Kansas City, Kansas City, Missouri, USA; d Children’s Hospital of Philadelphia, Perelman School of Medicine, Philadelphia, Pennsylvania, USA; e University of Texas Southwestern Medical Center, Dallas, Texas, USA; f Ann & Robert H. Lurie Children’s Hospital of Chicago, Chicago, Illinois, USA; g Cincinnati Children’s Hospital Medical Center, Cincinnati, Ohio, USA; h University of Cincinnati, Cincinnati, Ohio, USA; i Children’s Hospitals and Clinics of Minnesota, Minneapolis, Minnesota, USA; j Yale New Haven Children’s Hospital, Yale School of Medicine, New Haven, Connecticut, USA; k University Hospitals Cleveland Medical Center, Cleveland, Ohio, USA; l The Children’s Hospital at TriStar Centennial, Nashville, Tennessee, USA; m Sarah Cannon Research Institute, Nashville, Tennessee, USA; n MemorialCare Miller Children’s and Women’s Hospital Long Beach, Long Beach, California, USA; o Norton Children’s and University of Louisville School of Medicine, Louisville, Kentucky, USA; p Duke University Medical Center, Durham, North Carolina, USA; q Basilea Pharmaceutica International Ltd., Basel, Switzerland; r Astellas Pharma Global Development, Inc., Northbrook, Illinois, USA; s Weill Cornell Medicine, Cornell University, New York, New York, USA

**Keywords:** invasive fungal infections, isavuconazole, pediatric, population pharmacokinetics, triazole

## Abstract

Isavuconazole, administered as the water-soluble prodrug isavuconazonium sulfate, is a new triazole agent used to treat invasive fungal infections. This phase 1 study evaluated the pharmacokinetics (PK), safety, and tolerability of isavuconazole in 46 immunocompromised pediatric patients, stratified by age (1 to <6 [intravenous (i.v.) only], 6 to <12, and 12 to <18 years), receiving 10 mg/kg body weight (maximum, 372 mg) isavuconazonium sulfate either i.v. or orally. A population PK model using weight-based allometric scaling was constructed with the pediatric i.v. and oral data plus i.v. data from a phase 1 study in adults. The best model was a 3-compartment model with combined zero-order and first-order input, with linear elimination. Stepwise covariate modeling was performed in Perl-speaks-NONMEM version 4.7.0. None of the covariates examined, including age, sex, race, and body mass index, were statistically significant for any of the PK parameters. The area under the concentration–time curve at steady state (AUC_SS_) was predicted for pediatric patients using 1,000 Monte Carlo simulations per age cohort for each administration route. The probability of target attainment (AUC_SS_ range, 60 to 233 μg · h/ml) was estimated; this target range was derived from plasma drug exposures in adults receiving the recommended clinical dose. Predicted plasma drug exposures were within the target range for >80% and >76% of simulated pediatric patients following i.v. or oral administration, respectively. Intravenous and oral administration of isavuconazonium sulfate at the studied dosage of 10 mg/kg was well tolerated and resulted in exposure in pediatric patients similar to that in adults. (This study has been registered at ClinicalTrials.gov under identifier NCT03241550).

## INTRODUCTION

Invasive fungal infections are a major cause of morbidity and mortality in immunocompromised children (1). The increased use of curative intensive chemotherapeutic regimens and stem cell or solid organ transplants has expanded the pediatric population who are both at risk for, and suffer from, invasive fungal diseases due primarily to *Candida* spp., Aspergillus spp., and Mucorales (1, 2).

Compared with amphotericin B formulations, newer mold-active triazole antifungal agents have reduced nephrotoxicity and infusion-related side effects, and they are also available in oral formulations (3–5). However, high intrapatient and interpatient pharmacokinetic (PK) variability in children has made the use of these agents challenging (6–13). Isavuconazole (ISAV), which is administered as the water-soluble prodrug isavuconazonium sulfate, is an FDA- and European Medicines Agency-approved broad-spectrum triazole antifungal agent that has a favorable safety profile and demonstrated efficacy to treat invasive aspergillosis and other invasive mold infections, including mucormycosis, in adults (14).

Isavuconazonium sulfate may be administered intravenously (i.v.) or orally, with or without food, and the recommended adult dose for both routes is 372 mg (equivalent to 200 mg ISAV) every 8 h for 6 doses as a loading regimen, followed by a maintenance dose of 372 mg once daily (14). The PK of ISAV is well characterized in adults (15, 16), and dose-proportional and predictable PK has been demonstrated in adults in both clinical trials and real-world use (16, 17). ISAV plasma concentrations are higher after the loading regimen than at steady state and are near (within 90% of) steady state by day 7 of dosing (Astellas, data on file [see the “Data availability” paragraph in the Materials and Methods section]).

Clinical guidelines for the treatment of invasive fungal infections are similar for adult and pediatric patients (18–22). However, the PKs of other mold-active triazoles, such as voriconazole and posaconazole, differ between adults and children; specifically, children are less likely to attain plasma levels that have been associated with efficacy in adult studies (6, 10, 23–25). PK and safety studies following isavuconazonium sulfate administration are required to establish dosing guidelines for pediatric patients.

The aim of this open-label, phase 1 study was to evaluate the population PK, safety, and tolerability of ISAV following multiple doses of i.v. and oral isavuconazonium sulfate in pediatric patients at risk for invasive mycoses. The PK data were then used to establish a pediatric population PK model of ISAV.

(These data were presented in part as a poster at the 28th European Congress of Clinical Microbiology & Infectious Diseases [ECCMID], 21 to 24 April 2018, Madrid, Spain, and as an oral presentation at the 29th ECCMID, 13 to 16 April 2019, Amsterdam, Netherlands. An abstract was accepted by the 30th ECCMID, 18 to 21 April 2020, Paris, France.)

## RESULTS

### Disposition of patients.

Of 49 patients enrolled across both the i.v. and oral cohorts, two patients in the i.v. group and one patient in the oral group did not receive study drug; 46 patients were therefore included in the safety analysis set (see Fig. S1 in the supplemental material). Of these, 45 provided at least one valid plasma concentration measurement and were included in the PK analysis set, and 38 completed the study (Fig. S1).

### Demographics and baseline characteristics (safety analysis set).

Patients in the i.v. group (*n *=* *27) were predominantly male (74.1%) and white (66.7%), with age ranging from 1 to 17 years (Table 1). In the oral group (*n *=* *19), just over half of patients were female (52.6%) and the majority were white (78.9%), with age ranging from 6 to 17 years (Table 1). The most common underlying conditions were acute leukemia, neuroblastoma, and aplastic anemia (Table 1). Four (14.8%) patients in the i.v. cohort and three (15.8%) patients in the oral cohort had received a prior hematopoietic stem cell transplant. The majority (42/46, 91.3%) of patients had been or were receiving chemotherapy.

**TABLE 1:** Demographics and baseline characteristics (safety analysis set)^*a*^

Parameter	Data for i.v. cohort:					Data for oral cohort:		
	1 to <6 yrs (*n *=* *9)	6 to <12 yrs (*n *=* *8)		12 to <18 yrs (*n *=* *10)	Total (*n *=* *27)	6 to <12 yrs (*n *=* *9)	12 to <18 yrs (*n *=* *10)	Total (*n *=* *19)
Sex (*n* [%])								
Male	6 (66.7)	6 (75.0)		8 (80.0)	20 (74.1)	5 (55.6)	4 (40.0)	9 (47.4)
Female	3 (33.3)	2 (25.0)		2 (20.0)	7 (25.9)	4 (44.4)	6 (60.0)	10 (52.6)
Ethnicity (*n* [%])								
Hispanic or Latino	3 (33.3)	2 (25.0)		4 (44.4)	9 (34.6)	7 (77.8)	2 (20.0)	9 (47.4)
Not Hispanic or Latino	6 (66.7)	6 (75.0)		5 (55.6)	17 (65.4)	2 (22.2)	8 (80.0)	10 (52.6)
Missing	0	0		1	1	0	0	0
Race (*n* [%])								
White (including Hispanic)	5 (55.6)	7 (87.5)		6 (60.0)	18 (66.7)	8 (88.9)	7 (70.0)	15 (78.9)
Black	2 (22.2)	1 (12.5)		1 (10.0)	4 (14.8)	0	1 (10.0)	1 (5.3)
Asian	1 (11.1)	0		1 (10.0)	2 (7.4)	0	1 (10.0)	1 (5.3)
American Indian or Alaska native	0	0		0	0	1 (11.1)	0	1 (5.3)
Pacific Islander	1 (11.1)	0		0	1 (3.7)	0	0	0
Other	0	0		2 (20.0)	2 (7.4)	0	1 (10.0)	1 (5.3)
Median age (range [yrs])	3.0 (1–5)	9.5 (6–11)		14.5 (12–17)	10.0 (1–17)	10.0 (6–11)	14.5 (12–17)	12.0 (6–17)
Median wt (range [kg])	15.6 (10.9–19.1)	32.7 (18.6–67.4)		65.9 (42.4–103.5)	33.7 (10.9–103.5)	26.8 (18.3–50.1)	50.4 (37.9–92.8)	42.6 (18.3–92.8)
Median BMI (range [kg/m^2^])	16.2 (13.8–20.1)	17.1 (13.7–23.9)		24.3 (16.3–31.6)	18.5 (13.7–31.6)	16.7 (13.6–23.2)	19.8 (16.6–31.1)	18.7 (13.6–31.1)
Primary underlying condition (*n* [%])								
Acute myelogenous leukemia	4 (44.4)	2 (25.0)		4 (40.0)	10 (37.0)	4 (44.4)	2 (20.0)	6 (31.6)
Acute lymphoblastic leukemia	0	1 (12.5)		1 (10.0)	2 (7.4)	2 (22.2)	3 (30.0)	5 (26.3)
Neuroblastoma	3 (33.3)	1 (12.5)		0	4 (14.8)	0	0	0
Other solid tumor^*b*^	0	0		1 (10.0)	1 (3.7)	0	0	0
Aplastic anemia	0	2 (25.0)		1 (10.0)	3 (11.1)	1 (11.1)	0	1 (5.3)
Other^*c*^	2 (22.2)	2 (25.0)		3 (30.0)	7 (25.9)	2 (22.2)	5 (50.0)	7 (36.8)

a Safety analysis set included all patients who received ≥1 dose of the study drug. BMI, body mass index; i.v., intravenous.

b Metastatic Ewing’s sarcoma.

c Acute lymphoblastic leukemia relapse, acute myeloblastic leukemia, acute myeloid leukemia French-American-British classification M5, acute osteomyelitis, chronic granulomatous disease, cystic fibrosis, Fanconi anemia, hemophagocytic lymphohistiocytosis, idiopathic aplastic anemia, immune disorder, myelodysplastic syndrome, myelodysplastic syndrome with 5q deletion, myeloid sarcoma, X-linked adrenoleukodystrophy, severe combined immunodeficiency disease.

### Isavuconazonium sulfate administration (safety analysis set).

**(i) Intravenous cohorts.** For i.v. administration, all nine patients in the 1 to <6 years cohort and six of eight patients in the 6 to <12 years cohort weighed ≤37 kg and were dosed at 10 mg/kg body weight. The remainder (two patients in the 6 to <12 years cohort and all 10 in the 12 to <18 years cohort) weighed >37 kg and received a dose of 372 mg. The loading and maintenance dosing regimens are described in Materials and Methods. The duration of maintenance treatment ranged from 1 to 26 days (Table S1).

**(ii) Oral cohorts.** For oral dosing, six of nine patients in the 6 to <12 years cohort weighed ≤32 kg and were dosed at 10 mg/kg, while three in this cohort and all 10 in the 12 to <18 years cohort weighed >32 kg and received a dose of 372 mg. The loading and maintenance dosing regimens are described in Materials and Methods. The duration of maintenance treatment ranged from 2 to 25 days (Table S1).

**(iii) Concomitant medication use.** Concomitant medication use was as expected in this high-risk pediatric population. Most (31/46, 67.4%) patients had received previous systemic antifungal medications, and 15 (32.6%) patients received concomitant systemic antifungal agents (micafungin primarily, amphotericin B, or fluconazole; any triazoles were non-mold-active) during the treatment period.

### ISAV exposure (PK analysis set).

In the i.v. and oral cohorts, 26 and 19 patients, respectively, provided at least one blood sample for a total of 551 plasma samples (333 from the i.v. cohorts, 218 from the oral cohorts) for PK analysis.

**(i) Noncompartmental PK analysis.** In the i.v. cohorts, maximum plasma concentration (*C*_max_) and area under the concentration–time curve to the last measurable concentration (AUC_tau_) values on days 3 (not at steady state) and 7 in the 12 to <18 years cohort were generally lower than those in the 1 to <6 years and 6 to <12 years cohorts, but the ranges in individual values overlapped; median time to *C*_max_ (*T*_max_) was similar across the age cohorts (Table 2). In the oral cohorts, there was a tendency for the *C*_max_ and AUC_tau_ values on day 7 to be slightly lower in the 12 to <18 years cohort than in the 6 to <12 years cohort, but the ranges in individual values overlapped; the median *T*_max_ was similar in the two age cohorts (Table 2).

**TABLE 2:** Summary of plasma pharmacokinetic parameters derived from noncompartmental analysis (pharmacokinetic analysis set)^*a*^

Parameter	Data for i.v. cohort:			Data for oral cohort:	
	1 to <6 yrs (*n *=* *9)	6 to <12 yrs (*n *=* *8)	12 to <18 yrs (*n *=* *9)	6 to <12 yrs (*n *=* *9)	12 to <18 yrs (*n *=* *10)
Day 3 (±1)					
* C*_max_ (μg/ml)					
* n*	8	6	8	NA	NA
* *Mean (SD)	7.81 (0.83)	7.80 (1.64)	5.53 (2.32)		
* *Median (min–max)	7.96 (6.52–8.93)	8.11 (5.02–9.42)	5.20 (2.97–9.73)		
* *AUC_tau_ (μg · h/ml)					
* n*	8	6	8	NA	NA
* *Mean (SD)	112.0 (25.0)	102.0 (35.0)	70.1 (29.6)		
* *Median (min–max)	105.0 (79.9–157.0)	107.0 (58.8–156.0)	61.6 (41.8–132.0)		
* T*_max_ (h)					
* n*	8	6	8	NA	NA
* *Median (min–max)	1.11 (0.88–1.17)	1.08 (1.02–4.37)	1.11 (0.90–1.17)		
Day 7 (±1)					
* C*_max_ (μg/ml)					
* n*	9	8	7	9	8
* *Mean (SD)	7.31 (1.21)	6.78 (2.11)	5.02 (1.20)	6.04 (2.24)	5.03 (2.17)
* *Median (min–max)	7.31 (5.84–9.96)	6.97 (4.44–9.91)	5.65 (3.44–6.12)	5.78 (2.87–8.93)	5.43 (1.95–7.75)
* *AUC_tau_ (μg · h/ml)					
* n*	8	8	6	7	5
* *Mean (SD)	96.8 (47.3)	87.2 (33.2)	76.8 (20.5)	111.0 (50.2)	83.3 (33.4)
* *Median (min–max)	102.0 (43.0–179.0)	78.2 (56.0–144.0)	77.8 (54.1–103.0)	121.0 (48.6–185.0)	76.7 (37.6–127.0)
* T*_max_ (h)					
* n*	9	8	7	9	8
* *Median (min–max)	1.08 (1.03–1.35)	1.08 (1.02–1.22)	1.07 (1.02–1.20)	4.00 (1.98–6.08)	3.98 (3.05–8.03)

a Pharmacokinetic analysis set included all patients who received ≥1 dose of the study drug and had ≥1 plasma concentration measurement. AUC_tau_, area under the concentration–time curve over a dosing interval; *C*_max_, maximum plasma concentration; NA, not applicable; *T*_max_, time to maximum plasma concentration; SD, standard deviation.

**FIG 1:**
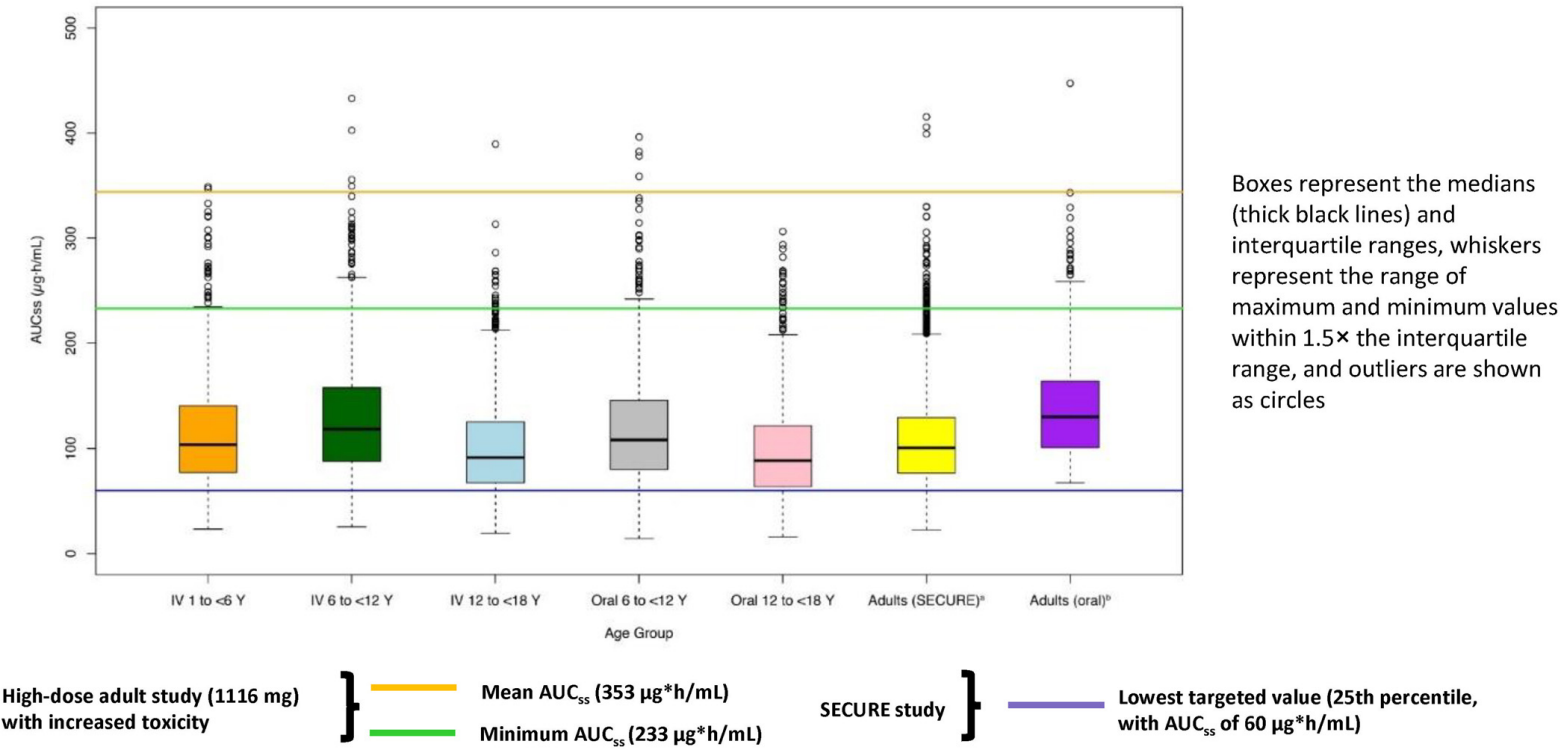
Comparison of predicted steady-state isavuconazole plasma drug exposures from simulated pediatric patients with observed adult exposures. ^a^SECURE data are derived from the exposure-response analysis of Desai et al. (30). ^b^Adult oral data are derived from an analysis that included adult patients from the phase 3 SECURE and VITAL studies who received oral administration (26). AUC_SS_, area under the concentration–time curve at steady state; i.v., intravenous; Y, years.

**FIG 2:**
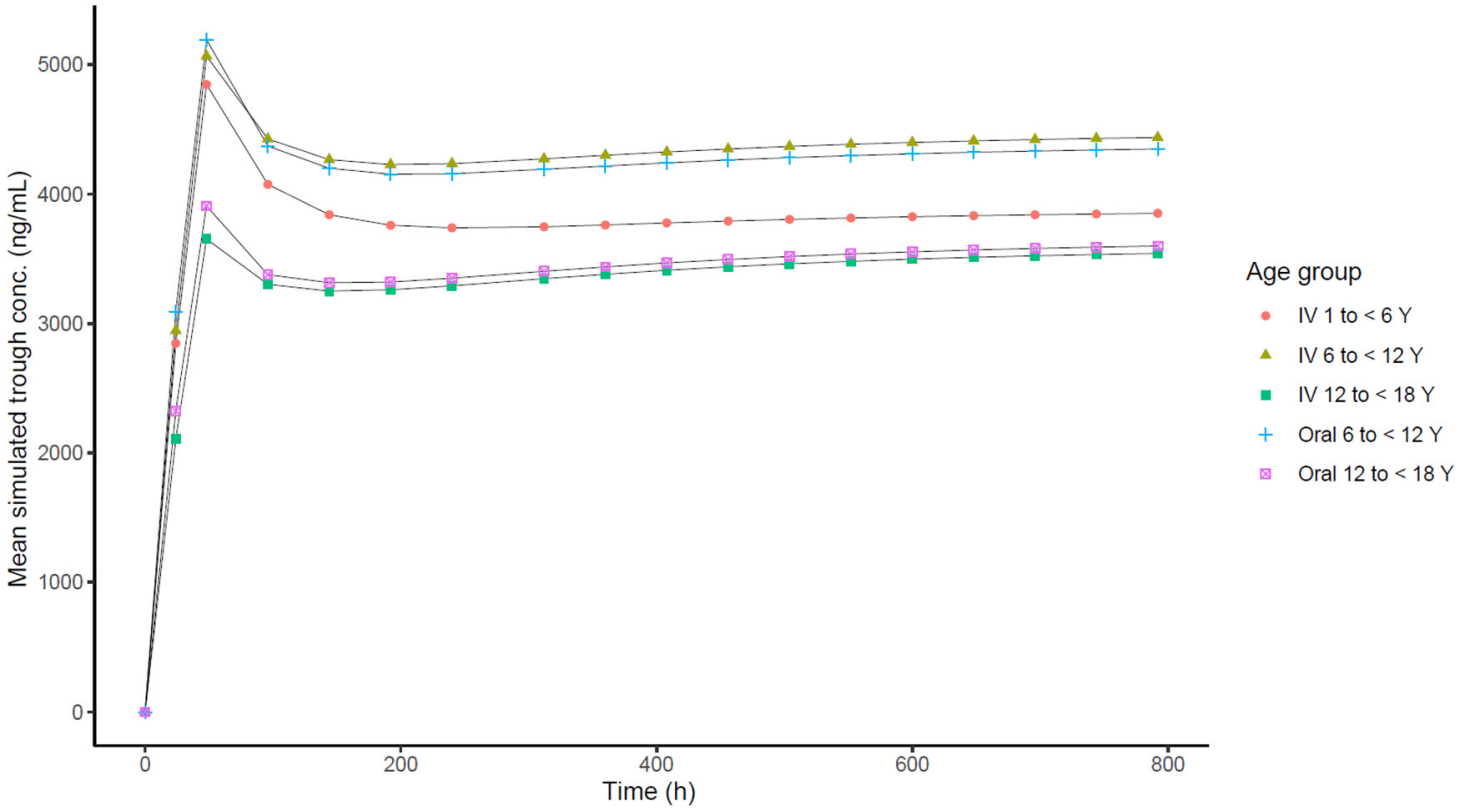
Comparison of mean simulated steady-state isavuconazole plasma drug trough concentrations for pediatric subjects by age group. i.v., intravenous; Y, years.

**(ii) Population PK model analysis.** The best model was a 3-compartment model with combined zero-order and first-order input, with linear elimination. None of the covariates examined (age, sex, race, body mass index [BMI], serum creatinine, alanine aminotransferase [ALT], aspartate aminotransferase [AST], total bilirubin, albumin, or alkaline phosphatase [AP]) were statistically significant for any of the PK parameters in the best model. The parameter estimates for the best model and the results for 1,000 bootstrap replicates are summarized in Table S2. The mean parameter estimates from bootstrapping were comparable to the parameter estimates from the best model. Just 4% of bootstrap runs failed in the best model. Goodness-of-fit plots for the best model are presented in Fig. S2. Based on the final model, predicted ISAV plasma drug exposures in simulated pediatric patients were similar following i.v. or oral administration (Fig. 1). Population concentrations were simulated to steady state for pediatric patients in all age groups. Simulated concentrations were similar within different age groups between i.v. and oral routes of administration (Fig. 2).

Following isavuconazonium sulfate dosing at 10 mg/kg up to a maximum 372 mg (corresponding to 5.38 mg/kg or maximum 200 mg dose of ISAV), predicted ISAV plasma drug exposures (AUC at steady state [AUC_SS_]) in simulated pediatric patients were similar to systemic ISAV plasma drug exposures from adults treated in the phase 3 SECURE (ClinicalTrials.gov, NCT00412893) and VITAL (ClinicalTrials.gov, NCT00634049) studies (Fig. 1) (16, 26). Predicted ISAV AUC_SS_ values for simulated pediatric patients were within the target exposure range of 60 to 233 μg · h/ml for >80% of simulated patients when isavuconazonium sulfate was administered i.v. and >76% of simulated patients when administered orally (Table 3). With either i.v. or oral administration, a higher proportion of simulated pediatric patients aged 12 to <18 years had predicted ISAV AUC_SS_ values below 60 μg · h/ml compared with younger cohorts.

**TABLE 3:** Proportion of pediatric patients with predicted AUC_SS_ values above or below the specified adult AUC values (pharmacokinetic analysis set)^*a*^

Measurement	Data for i.v. cohort:			Data for oral cohort:	
	1 to <6 yrs (*n *=* *9)	6 to <12 yrs (*n *=* *8)	12 to <18 yrs (*n *=* *9)	6 to <12 yrs (*n *=* *9)	12 to <18 yrs (*n *=* *10)
Patients below the target range (60 μg · h/ml)^*b*^ (%)	11.4	7.2	18.0	9.3	21.6
Patients within target range (60–233 μg · h/ml)^*c*^ (%)	85.7	86.8	80.2	87.1	76.5

a Pharmacokinetic analysis set included all patients who received ≥1 dose of the study drug and had ≥1 plasma concentration measurement.

b Delineation of the 25th percentile of AUC_SS_ from the phase 3 SECURE study in adults (30).

c Upper limit was the minimum AUC from 0 to 24 h (AUC_0–24_) from a phase 1 safety study in adults using a supratherapeutic dose of isavuconazonium sulfate (1,116 mg) (27). AUC_SS_, area under the isavuconazole concentration–time curve at steady state (predicted values); i.v., intravenous.

Calculated ISAV AUC_SS_ values were significantly below the safety threshold reported previously following supratherapeutic dosing in a safety study in adults (Fig. 1) (27).

### Safety.

**(i) Adverse events.** There were no deaths during the study in either the i.v. or oral cohorts.

Treatment-emergent adverse events (TEAEs) were common in both the i.v. (25/27; 92.6%) and oral (18/19; 94.7%) cohorts. The most common TEAEs in both cohorts were pyrexia and gastrointestinal disturbances, mainly diarrhea in the i.v. cohort (9/27; 33.3%) and vomiting in the oral cohort (7/19; 36.8%). TEAEs at least possibly related to the study drug were common (17 in 10 patients in the i.v. cohort and 19 in 10 patients in the oral cohort). Study drug-related serious TEAEs were rare (diarrhea and pyrexia in the i.v. cohort, three each) (Table 4; Tables S3 and S4).

**TABLE 4:** Summary of treatment-emergent adverse events (safety analysis set)^*a*^

Event	Data for i.v. cohort:				Data for oral cohort:		
	1 to <6 yrs (*n *=* *9)	6 to <12 yrs (*n *=* *8)	12 to <18 yrs (*n *=* *10)	Total (*n *=* *27)	6 to <12 yrs (*n *=* *9)	12 to <18 yrs (*n *=* *10)	Total (*n *=* *19)
Any TEAE (*n* [%])	7 (77.8)	8 (100.0)	10 (100.0)	25 (92.6)	9 (100.0)	9 (90.0)	18 (94.7)
Any serious TEAE (*n* [%])^*b*^	3 (33.3)	4 (50.0)	5 (50.0)	12 (44.4)	5 (55.6)	3 (30.0)	8 (42.1)
Any TEAE leading to discontinuation of study drug	0	1 (12.5)	1 (10.0)	2 (7.4)	3 (33.3)	1 (10.0)	4 (21.1)
Any drug-related TEAE (*n* [%])^*c*^	3 (33.3)	3 (37.5)	4 (40.0)	10 (37.0)	4 (44.4)	6 (60.0)	10 (52.6)
Any drug-related serious TEAE (*n* [%])^*b*^^,^^*c*^	0	0	1 (10.0)^*d*^	1 (3.7)	1 (11.1)^*e*^	0	1 (5.3)
Any drug-related TEAE leading to discontinuation of study drug (*n* [%])^*c*^	0	1 (12.5)^*f*^	1 (10.0)^*d*^	2 (7.4)	3 (33.3)^*e*^^,^^*g*^^,^^*h*^	0	3 (15.8)
TEAEs occurring i*n* ≥35% of patients in any treatment cohort (*n* [%])							
Pyrexia	4 (44.4)	5 (62.5)	5 (50.0)	14 (51.9)	5 (55.6)	2 (20.0)	7 (36.8)
Mucosal inflammation	5 (55.6)	4 (50.0)	3 (30.0)	12 (44.4)	0	1 (10.0)	1 (5.3)
Diarrhea	2 (22.2)	4 (50.0)	3 (30.0)	9 (33.3)	2 (22.2)	1 (10.0)	3 (15.8)
Vomiting	2 (22.2)	0	0	2 (7.4)	3 (33.3)	4 (40.0)	7 (36.8)
Pain in extremity	1 (11.1)	1 (12.5)	4 (40.0)	6 (22.2)	1 (11.1)	1 (10.0)	2 (10.5)
Dysuria	0	3 (37.5)	1 (10.0)	4 (14.8)	0	0	0

a Safety analysis set included all patients who received ≥1 dose of the study drug. i.v., intravenous; TEAE, treatment-emergent adverse event.

b Includes serious TEAEs upgraded by the sponsor based on review of the sponsor’s list of Always Serious terms.

c Reasonable possibility that the event may have been caused by the study drug as assessed by the investigator; if relationship was missing, the event was considered drug-related.

d Prolonged QT interval in a 16-year-old girl (day –3 [baseline], QTc = 479 msec; day 7, QTc = 484 msec).

e Four serious TEAEs (tachycardia, nausea, vomiting, and pyrexia) occurred in a single patient; three of these (nausea, vomiting, and pyrexia) resulted in discontinuation of the study drug.

f Increased hepatic enzymes (reported as severe).

g Increased transaminases.

h Upper abdominal pain.

No invasive fungal infections were reported during the study. Two noninvasive fungal infections were reported, both oral candidiasis, one in the i.v. 1 to <6 years cohort and one in the oral 6 to <12 years cohort. Both patients were treated with oral nystatin. Four cases of microbiologically documented serious bacterial infections were reported in the i.v. and oral cohorts. A case of serious bacteremia was reported in the i.v. 6 to <12 years cohort, while single cases of serious bacterial pneumonia and serious staphylococcal bacteremia were reported in the oral 6 to <12 years cohort, and a case of serious Clostridium difficile infection was reported in the oral 12 to <18 years cohort.

**(ii) Laboratory parameters, vital signs, and electrocardiogram results.** Apart from the increases in transaminases described above, there were no clinically significant changes in laboratory parameters, blood pressure, or pulse rate, as judged by the investigator. Apart from the serious TEAE of prolonged QT interval possibly related to the study drug in a patient in the i.v. cohort and a TEAE of conduction disorder (mild severity) possibly related to the study drug in a patient in the oral cohort, there were no other clinically significant electrocardiogram (ECG) abnormalities.

## DISCUSSION

This is the first study to formally investigate the PK and safety of ISAV when administered as i.v. or oral isavuconazonium sulfate in pediatric patients. Drug disposition was best described by a 3-compartment model with combined zero-order and first-order input, with linear elimination of ISAV. Allometric scaling based on body weight was used to scale size-related changes in clearance and volume of distribution. Based on this model, a 10-mg/kg (maximum 372-mg) dose of isavuconazonium sulfate (5.38 mg/kg or maximum 200 mg of ISAV) provided plasma drug exposures within the target range (AUC_SS_, 60 to 233 μg · h/ml) for >80% of simulated pediatric patients with i.v. dosing and >76% of simulated pediatric patients with oral dosing. Of the small proportion of predicted pediatric plasma drug exposures not within the target range, the majority were below the lower threshold. We defined the target drug exposure of AUC_SS_ 60 to 233 μg · h/ml after careful analysis of the adult ISAV plasma drug exposures, as well as the efficacy and safety associated with these plasma drug exposures in the SECURE and VITAL studies and safety associated with a supratherapeutic isavuconazonium sulfate dose (1,116 mg) in an adult phase 1 study (15, 27–30). As a formal exposure-response analysis in the adult population from the SECURE study did not find a plasma drug exposure that clearly delineated patients who were successfully versus unsuccessfully treated (30), the lower bound of the target plasma drug exposure range for pediatric patients was set at the 25th percentile limit of the adult plasma drug exposure range in the SECURE study. Predicted plasma drug exposures for pediatric patients, irrespective of route of administration and age group, were comparable to the range of plasma drug exposures from the SECURE study, which was conducted in adult patients with infections caused by Aspergillus spp. and other filamentous fungi, where the routes of administration were both i.v. and oral (16). The model parameter estimates for clearance were nearly identical to the population mean estimates reported for adults from the SECURE and VITAL studies (15, 16, 26). Predicted plasma drug exposures for pediatric patients were also within the range of plasma drug exposures for adults from the SECURE and VITAL studies who received oral administration (26). At the doses administered in this study, the predicted ISAV exposures for pediatric patients were well below the ISAV exposures observed in a previous study of adults given a supratherapeutic dose of isavuconazonium sulfate (1,116 mg), which was associated with an increased occurrence of adverse events (AEs) (27).

None of the covariates analyzed, including BMI, significantly affected exposure or target attainment of ISAV when isavuconazonium sulfate was administered to pediatric patients at a dose of 10 mg/kg (maximum dose of 372 mg). Nonetheless, high body weight and BMI have been associated with higher ISAV clearance rate in adults with invasive fungal disease (15). In our analysis, a higher proportion of predicted pediatric plasma drug exposures falling below the lower target threshold (AUC_SS_, <60 μg · h/ml) after i.v. or oral administration was in the 12 to <18 years age group compared with younger groups. This may reflect a greater proportion of older patients weighing >37 kg (i.v.) or >32 kg (oral) and receiving the adult dose of 372 mg and our observation that the 12 to <18 years cohorts received a lower mean dosage (mg/kg) than younger cohorts. Similarly, in our noncompartmental PK analysis in pediatric patients, *C*_max_ and AUC_tau_ values on days 3 (i.v.) and 7 (i.v. and oral) in the 12 to <18 years cohorts tended to be lower than in the younger cohorts. A significant proportion of patients aged 12 to <18 years exhibited AUC_SS_ of <60 μg · h/ml.

Our observations suggest there may be a possibility of underdosing in pediatric patients with body weight of >37 kg, although the lower end of the target exposure range adopted in this study has not been tied to efficacy data. Children with high BMIs (>30 kg/m^2^) may have insufficient dosing at 372 mg of isavuconazonium sulfate because of several factors, including increased clearance and volume of distribution. Monitoring of plasma AUC in such patients may be warranted to ensure that the steady-state value exceeds 60 μg · h/ml. In such cases, a higher dose may be necessary to achieve target exposure. Further studies are needed to validate approaches to therapeutic-drug monitoring (TDM) in immunocompromised pediatric patients receiving ISAV. The critical objective of TDM of ISAV in pediatric patients would be to ensure adequate circulating plasma concentrations for treatment of invasive fungal infections. This contrasts with the TDM objectives for voriconazole, where adjustment of dosage is needed for safety and tolerability as well as efficacy (31).

There are limits to the generalizability of the findings of this study to other pediatric populations. This study is intended to test primarily the acute safety of a small number of patients and characterize the PKs. While the cohort size was fit for purpose, it limits the ability to make conclusions for a broader population beyond the objectives of this study. The patients in our pediatric study were predominantly white and, while visual inspection showed no correlation between race and clearance, limited numbers from nonwhite racial groups precluded analysis of the effect of race on ISAV exposure. In adults, clearance of ISAV is lower in Asians than in Caucasians, resulting in a 40% greater exposure for a given dose in Asian patients compared with Caucasian patients (16). The reason for the lower clearance in Asians has not been established, although it is unlikely to be due to CYP2D6 or CYP2C19, as ISAV is not a substrate for either isoenzyme (16). It may be related to allelic polymorphisms in *CYP3A4* and *CYP3A5*, which previously were not considered to be active in altered drug metabolism (32–34). For example, a study of the metabolism of voriconazole in Chinese patients suggests that the rs4646437 T allele of *CYP3A4* is a significant factor in altered metabolism and serum concentrations (35). Further population-based pharmacogenomic studies of ISAV in Asian populations are warranted to better understand the potential role of the allelic polymorphisms of *CYP3A4* and *CYP3A5* in drug exposure.

Isavuconazonium sulfate at the studied doses was generally well tolerated in pediatric patients, with an overall safety profile similar to that observed in adults. No new or unexpected safety signals were identified. As most participants were oncology patients entering the neutropenic phase of their chemotherapeutic regimens, the development of pyrexia, mucosal inflammation, diarrhea, and vomiting would be expected. At the same time, the development of elevated transaminases during the course of antineoplastic therapy may be related to cytotoxic therapy, hepatic graft versus host disease, or the hepatotoxic class effect of antifungal triazoles. No obvious differences in frequency and types of TEAEs were observed among age cohorts.

In conclusion, a majority of pediatric patients receiving isavuconazonium sulfate 10 mg/kg (up to 372 mg) daily are predicted to achieve plasma drug exposure similar to that observed in adults in phase 3 efficacy trials. This level of plasma drug exposure in adults was shown to be sufficient for treating pathogenic mold infections, although no statistically significant exposure/efficacy relationship was found. Drug exposure, however, is an important component of the pharmacodynamic index (AUC:MIC) shown to drive efficacy. As both i.v. and oral administration of isavuconazonium sulfate were well tolerated and resulted in similar exposures in pediatric patients, the route of administration may be switched between i.v. and oral as needed.

## MATERIALS AND METHODS

### Study design and participants.

This was an open-label, phase 1, multicenter, noncomparative study conducted in the United States. The study was conducted in accordance with the protocol, Good Clinical Practice, the International Council for Harmonisation (ICH) guidelines, applicable local regulations and guidelines governing clinical study conduct, and the ethical principles that have their origin in the Declaration of Helsinki. Witnessed written informed consent (and assent where appropriate) was obtained from all patients prior to all study procedures.

The study was performed in two parts; the first part consisted of three i.v. age cohorts (1 to <6 years, 6 to <12 years, and 12 to <18 years) at 11 centers, and the second consisted of two oral age cohorts (6 to <12 years and 12 to <18 years) at 12 centers. Patients <6 years of age were excluded from the oral group due to the size of the capsule.

The study recruited male and female patients identified by the treating physician as candidates who could benefit from systemic antifungal prophylaxis, primarily due to hematological malignancy, including hematological stem cell transplant recipients who are at high risk for invasive fungal disease (Table 1). The main inclusion and exclusion criteria are summarized in Table S1.

### Dose selection and treatment.

Patients in the i.v. cohorts received an i.v. isavuconazonium sulfate infusion (Cresemba; Astellas Pharma US, Inc., Northbrook, IL, USA) administered over 1 h. Patients in the oral cohorts received a novel isavuconazonium sulfate 74.5-mg oral capsule equivalent to 40 mg of ISAV; this oral capsule is smaller than that used in adults, which contains 186 mg of isavuconazonium sulfate (equivalent to 100 mg of ISAV). The chosen dose was intended to generate plasma drug exposures in the pediatric population that were consistent with the plasma drug exposures observed in adults receiving the recommended clinical dose; specifically, the target plasma ISAV exposure range (AUC_SS_) was 60 to 233 μg · h/ml. The lower limit of this range delineated the 25th percentile of AUC_SS_ from the phase 3 SECURE study that supported the indications for invasive aspergillosis and mucormycosis (30); the upper limit of this range was the minimum AUC from 0 to 24 h (AUC_0–24_) from an adult safety study that used a supratherapeutic dose (1,116 mg) to evaluate the risk of prolonged QT interval (27).

Population PK modeling using allometric scaling was performed to simulate pediatric exposure and establish a pediatric dose. Patients received a loading regimen of isavuconazonium sulfate (i.v. or oral depending on the cohort), which consisted of a target dose of 10 mg/kg (to a maximum of 372 mg) every 8 h for 6 doses, followed by a maintenance dose of 10 mg/kg (to a maximum of 372 mg) once daily for up to 26 additional days. The cutoff body weight of 37 kg used to determine the dose (≤37 kg, 10 mg/kg; >37 kg, 372 mg) in the i.v. part of the study was changed in the oral part to 32 kg for greater dosing accuracy due to fixed capsule strengths.

### Concomitant medications.

Treatment with concomitant drugs that are strong inhibitors or inducers of CYP3A4 (e.g., ketoconazole) was prohibited. Concurrent drugs that are CYP3A4 substrates with a narrow therapeutic range were prohibited or reduced in dose. Concomitant sirolimus, atorvastatin, cyclosporine, tacrolimus, midazolam, bupropion, mycophenolate mofetil, and digoxin could be used with caution. Concomitant use of systemic triazole antifungal agents was prohibited.

### PK sample collection.

**(i) Intravenous cohorts.** Plasma PK profiles were derived from multiple blood samples collected on days 3, 7, and 28 or end-of-treatment (EOT) visit (or within 2 days prior to the last dose). Serial blood samples (approximately 1 ml) were collected in potassium ethylenediaminetetraacetic acid Vacutainer tubes within 15 min prior to the start of the i.v. infusion, within 5 min of infusion completion, and within 4 to 8, 8 to 12, and 16 to 24 h after the start of infusion. If the patient was able to provide PK samples beyond day 7, a trough sample was taken weekly (±1 day), within 15 min prior to the start of that day’s infusion, through to day 28 or EOT.

**(ii) Oral cohorts.** Serial blood samples (approximately 1 ml) were collected ≤1 h before the first dose on days 2, 3, 5, 14 (±2 days), 21 (±2 days), and 28 (±2 days). In addition, plasma PK profiles were derived from multiple blood samples collected on day 7 (±1 day), with samples taken predose, and at 1, 2, 3, 4 (±10 min), 6 (±30 min), 8 (±30 min), and 24 h after study drug administration; the 24 h sample was taken ≤1 h before the next dose.

### Bioanalytical methods.

All blood samples were processed within 1 h after collection, and plasma was stored at or below −20°C until analysis. ISAV concentrations in plasma samples were measured using liquid chromatography-tandem mass spectrometry (LC-MS/MS) at Pharmaceutical Product Development, LLC (Middleton, WI, USA), as described in full previously (15). The lower limit of quantification was 100 ng/ml.

### Pharmacokinetic analysis.

This was a phase 1 study and the first pediatric PK study. Therefore, to characterize the pediatric PK profile fully, we first performed noncompartmental analyses. However, since these full PK profiles were collected earlier during ISAV prophylaxis administration, sparse sampling was collected later in the course of isavuconazole administration to build the population PK model and better characterize steady-state concentrations. These later data were then used for comparisons to adult data, the covariate analysis, and dose selection for further studies in the pediatric population.

### Noncompartmental PK parameters.

The parameters reported from observed data on day 3 (i.v. only) and day 7 were *C*_max_, AUC_tau_, and *T*_max_. The derived parameters were clearance, volume of distribution at steady state, AUC_SS_, and half-life.

### Population PK model.

A pediatric population PK model was constructed using the pediatric i.v. data, the pediatric oral data, and i.v. data from a phase 1 study in adults (36). The model included allometric scaling based on body weight to scale size-related changes in clearance and volume of distribution in pediatric patients, using the following equation:

$$P_{\text{pediatric}}=P_{\text{adults}}\text{ *}{(\frac{\mathrm{WT}}{70})}^{x}$$

where *P* is the parameter of interest (clearance or volume of distribution), *WT* is the weight in kg for the individual pediatric patient, and *x* for the relevant allometric component. The following covariates were examined for significant effects on clearance and volume of distribution: age, sex, race, BMI, serum creatinine, and liver assessments (ALT, AST, total bilirubin, albumin, and AP). Stepwise covariate modeling was performed in Perl-speaks-NONMEM version 4.7.0 (https://uupharmacometrics.github.io/PsN) by forward inclusion and backward elimination. Internal validation of the best model was performed using a nonparametric bootstrap procedure.

The model was used to simulate pediatric plasma drug exposures after i.v. and oral administration. Monte Carlo simulations (1,000 simulations per age cohort for each administration route) were performed in NONMEM version 7.3 (Icon Development Solutions, Ellicott City, MD, USA), and AUC_SS_ was calculated for each simulated pediatric patient in Phoenix WinNonlin version 6.4 (Certara, Princeton, NJ, USA). The percentage of simulated pediatric patients within the target exposure range, by age and route of administration, was calculated.

### Safety and tolerability assessments.

Safety assessments included AEs, vital signs, laboratory assessments, and ECG. AEs, defined as any untoward medical occurrence, were assessed throughout. TEAEs were defined as an AE starting after administration of the study drug through follow-up of 30 ± 2 days from day 28 or EOT. A serious AE was defined as an AE that resulted in death; was life-threatening; caused persistent or significant disability/incapacity, a congenital abnormality, or a birth defect; led to hospitalization or prolongation of hospitalization; or was another medically important event that may have jeopardized the patient or required intervention to prevent one of the other outcomes listed in the definition above. The investigator evaluated relatedness of AEs to the study drug (not related, possible, or probable), the severity of AEs, and resolution of AEs.

For the oral cohorts, vital signs were measured predose on dosing days if patients were hospitalized. For the i.v. cohorts, vital signs were measured ≤1 h prior to and approximately 1 h after the end of each infusion. Blood samples for clinical laboratory assessments were obtained at screening (as close to day 1 as possible if multiple samples were drawn) and on days 3 (±2 days), 7 (±2 days), 14 (±2 days), 21 (±2 days), and 28 or EOT (±2 days). For the i.v. cohorts, a 12-lead ECG was performed at screening, prior to dosing on days 1 and 14 (±2 days), at the end of infusion on day 7 (±2 days), and on day 28 or at EOT if before day 28. For the oral cohorts, a 12-lead ECG was performed at screening and prior to dosing on days 1, 7, 14, and 28 or EOT.

### Statistical analyses.

No formal sample size calculation was performed; the number of patients planned for the clinical study was considered sufficient to achieve the clinical study objectives. The safety analysis set consisted of all patients who took at least one dose of the study drug. The PK analysis set consisted of all patients who took at least one dose of the study drug and had at least one plasma concentration measurement. Data were summarized using descriptive statistics with no imputation of missing data.

### Data availability.

Researchers may request access to anonymized participant-level data, trial-level data, and protocols from Astellas-sponsored clinical trials at www.clinicalstudydatarequest.com. For the Astellas criteria on data sharing see https://clinicalstudydatarequest.com/Study-Sponsors/Study-Sponsors-Astellas.aspx.
