# Supplementary material for: Safety, Tolerability, and Population Pharmacokinetics of Intravenous and Oral Isavuconazonium Sulfate in Pediatric Patients
Source: Antimicrob Agents Chemother. 2021 Jul 16;65(8):e00290-21. doi: 10.1128/AAC.00290-21 (PMC8284446; doi:10.1128/AAC.00290-21)
Supplement: Supplemental file 1 — Supplemental figures and tables. Download AAC00290-21_Supp_1_seq4.pdf, PDF file, 0.4 MB [file aac00290-21_supp_1_seq4.pdf]

# Supplemental material

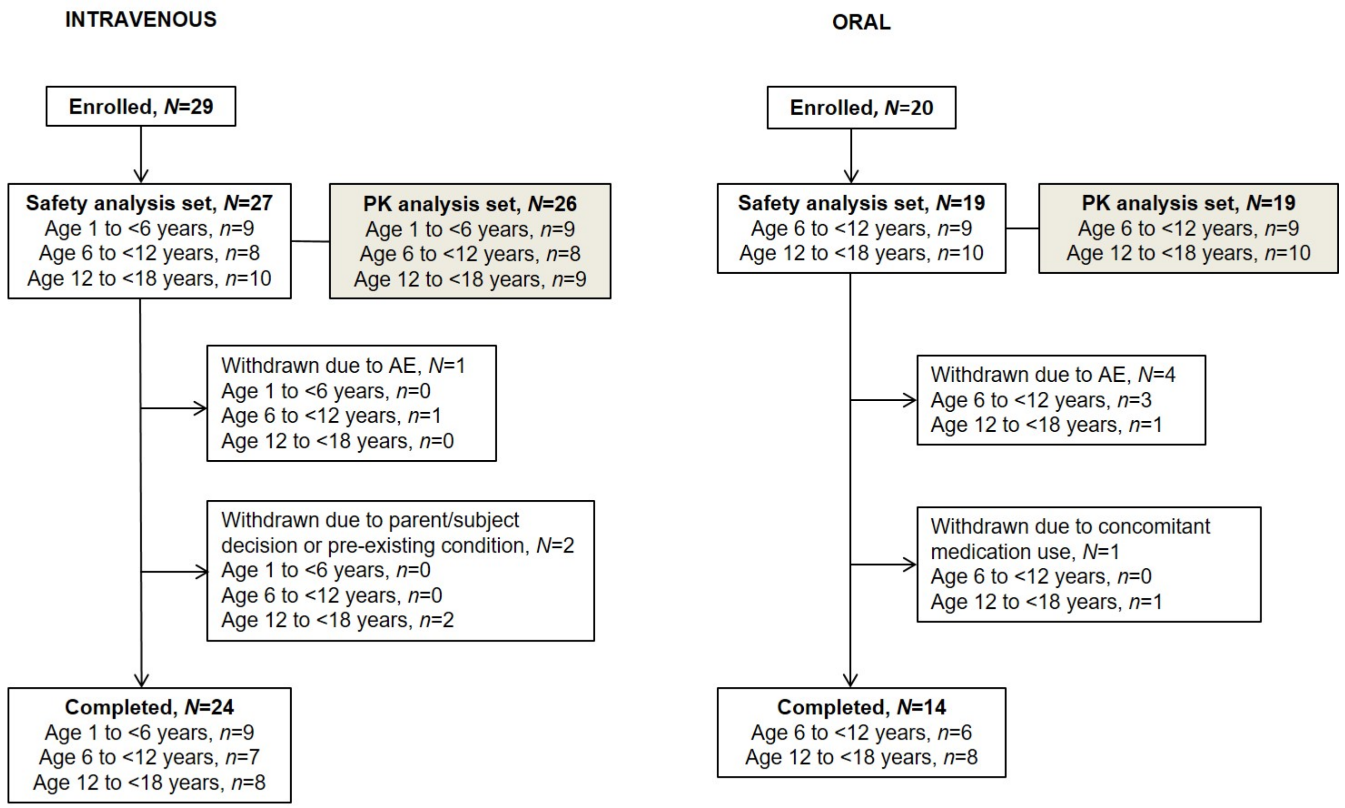


**FIG S1** Study flow diagram. PK analysis set included all enrolled patients who received ≥1 dose of study drug and who had at least one plasma concentration measurement. Safety analysis set included all enrolled patients who received ≥1 dose of study drug. AE, adverse event; PK, pharmacokinetic.

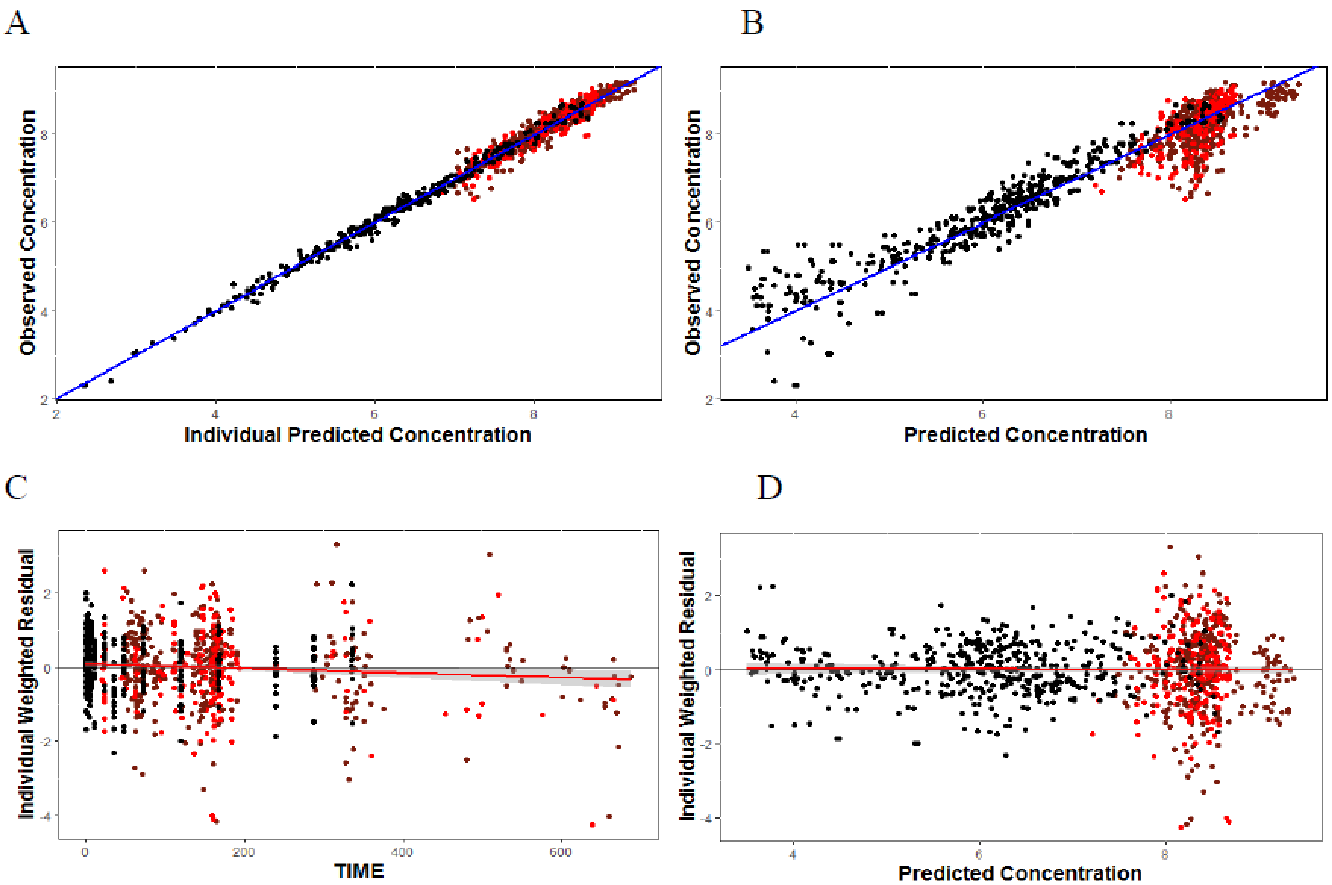


**FIG S2** Goodness-of-fit plots for the best population pharmacokinetic model. (A) Log of individual predicted concentrations versus log of observed concentrations. (B) Log of predicted concentrations versus log of observed concentrations. (C) Plot of individual weighted residual versus time. (D) Plot of individual weighted residual versus log of predicted concentrations. Black circles represent adult data, red circles represent pediatric data.

**TABLE S1** Treatment duration and daily dose of isavuconazonium sulfate (safety analysis set[a])

| | IV cohort | | | Oral cohort | |
|---|---|---|---|---|---|
| | **1 to <6 yrs (*n* = 9)** | **6 to <12 yrs (*n* = 8)** | **12 to <18 yrs (*n* = 10)** | **6 to <12 yrs (*n*=9)** | **12 to <18 yrs (*n*=10)** |
| Duration of loading phase, days[b] | | | | | |
| Mean (SD) | 2.9 (0.3) | 2.9 (0.4) | 2.7 (0.7) | 2.7 (0.5) | 2.8 (0.4) |
| Median (range) | 3.0 (2–3) | 3.0 (2–3) | 3.0 (1–3) | 3.0 (2–3) | 3.0 (2–3) |
| Duration of maintenance phase, days | | | | | |
| Mean (SD) | 12.0 (7.6) | 15.3 (8.9) | 7.8 (6.0) | 15.3 (8.2) | 9.6 (6.9) |
| Median (range) | 11.0 (4–26) | 12.5 (5–26) | 5.0 (1–21)[c] | 17.0 (4–25) | 8.0 (2–23) |
| Loading phase daily dose, mg/kg | | | | | |
| Mean (SD) | 21.9 (3.4) | 19.9 (5.1) | 11.7 (4.4) | 22.8 (5.5) | 16.5 (6.7) |
| Median (range) | 20.0 (20–30) | 20.0 (11–30) | 11.3 (5–18) | 23.0 (15–31) | 14.9 (8–28) |
| Maintenance phase daily dose, mg/kg | | | | | |
| Mean (SD) | 10.3 (0.8) | 9.3 (1.6) | 5.9 (1.6) | 9.8 (1.7) | 7.3 (2.0) |

| | | | | | |
|---|---|---|---|---|---|
| Median (range) | 10.0 (10–13) | 10.0 (6–10) | 5.9 (4–9) | 9.8 (7–12) | 7.4 (4–10) |

[a]Safety analysis set included all patients who received ≥1 dose of study drug.

[b]Depending on when dosing was started on day 1, the loading phase could end on day 2 or day 3.

[c]One patient withdrew consent after one dose due to a rash; IV, intravenous; SD, standard deviation.

**TABLE S2** Parameter estimates for the best population pharmacokinetic model

| Parameter | Units | Value | SE | % RSE | Bootstrap mean | Bootstrap 95% CI |
|---|---|---|---|---|---|---|
| CL | L/h | 2.55 | 0.156 | 6 | 2.55 | 2.26–2.83 |
| $V_2$ | L | 17.80 | 1.33 | 7 | 17.49 | 12.95–21.23 |
| $Q_3$ | L/h | 30.30 | 2.81 | 9 | 31.81 | 25.04–45.07 |
| $V_3$ | L | 26.00 | 2.60 | 10 | 26.67 | 20.83–34.67 |
| $Q_4$ | L/h | 24.50 | 2.58 | 11 | 24.41 | 20.11–28.69 |
| $V_4$ | L | 254.0 | 15.5 | 6 | 252.9 | 225.10–280.78 |
| $K_a$ | h | 0.162 | 0.023 | 14 | 0.162 | 0.122–0.217 |
| F1 | | 0.95 | 0.071 | 7 | 0.942 | 0.804–1.08 |
| Variability (%) | | | | | | |
| CL | | 45.16 | 0.034 | 17 | 44.60 | 36.6–51.9 |
| $V_4$ | | 68.11 | 0.128 | 28 | 67.82 | 36.4–83.6 |

| | | | | | |
|---|---|---|---|---|---|
| $Q_4$ | 45.71 | 0.052 | 25 | 45.49 | 36.6–54.77 |
| $V_3$ | 43.24 | 0.141 | 61 | 42.54 | 14.14–65.95 |
| Residual error $\sigma^2$ | 40.86 | 0.00934 | 6 | 40.62 | 38.47–42.77 |

CI, confidence interval; CL, clearance; F1, bioavailability; $K_a$, absorption rate constant; $Q_3$ and $Q_4$, inter-compartmental clearance values; % RSE, percent relative standard error; SE, standard error of the estimate; $V_2$, volume of distribution of central compartment; $V_3$ and $V_4$, volume of distribution of peripheral compartments.

**TABLE S3** Study drug-related treatment-emergent adverse events by MedDRA preferred term (safety analysis set[a])[b]

| Number (%) of patients | IV cohort | | | | Oral cohort | | |
|---|---|---|---|---|---|---|---|
| | **1 to <6 yrs (*n* = 9)** | **6 to <12 yrs (*n* = 8)** | **12 to <18 yrs (*n* = 10)** | **Total (*n* = 27)** | **6 to <12 yrs (*n* = 9)** | **12 to <18 yrs (*n* = 10)** | **Total (*n* = 19)** |
| **Overall** | **3 (33.3)** | **3 (37.5)** | **4 (40.0)** | **10 (37.0)** | **4 (44.4)** | **6 (60.0)** | **10 (52.6)** |
| Diarrhea | 1 (11.1) | 0 | 0 | 1 (3.7) | 1 (11.1) | 1 (10.0) | 2 (10.5) |
| Nausea | 0 | 0 | 1 (10.0) | 1 (3.7) | 2 (22.2) | 0 | 2 (10.5) |
| Vomiting | 0 | 0 | 0 | 0 | 2 (22.2) | 1 (10.0) | 3 (15.8) |
| Abdominal pain upper | 0 | 0 | 0 | 0 | 1 (11.1) | 0 | 1 (5.3) |
| Graft versus host disease | 0 | 1 (12.5) | 0 | 1 (3.7) | 0 | 0 | 0 |
| Pyrexia | 0 | 0 | 0 | 0 | 1 (11.1) | 0 | 1 (5.3) |
| Infusion-related reaction | 2 (22.2) | 0 | 0 | 2 (7.4) | NA | NA | NA |

| | | | | | | | |
|---|---|---|---|---|---|---|---|
| Procedural nausea | 2 (22.2) | 0 | 0 | 2 (7.4) | 0 | 0 | 0 |
| Procedural vomiting | 2 (22.2) | 0 | 0 | 2 (7.4) | 0 | 0 | 0 |
| ECG QT prolonged | 0 | 0 | 1 (10.0) | 1 (3.7) | 0 | 0 | 0 |
| Cardiac murmur | 0 | 1 (12.5) | 0 | 1 (3.7) | 0 | 0 | 0 |
| Conduction disorder | 0 | 0 | 0 | 0 | 1 (11.1) | 0 | 1 (5.3) |
| Tachycardia | 0 | 0 | 0 | 0 | 1 (11.1) | 0 | 1 (5.3) |
| Hepatic enzyme increased | 0 | 1 (12.5) | 0 | 1 (3.7) | 0 | 0 | 0 |
| ALT increased | 0 | 0 | 0 | 0 | 1 (11.1) | 0 | 1 (5.3) |
| AST increased | 0 | 0 | 0 | 0 | 1 (11.1) | 0 | 1 (5.3) |
| Weight decreased | 0 | 0 | 0 | 0 | 0 | 1 (10.0) | 1 (5.3) |
| Fluid imbalance | 0 | 1 (12.5) | 0 | 1 (3.7) | 0 | 0 | 0 |
| Headache | 0 | 0 | 0 | 0 | 1 (11.1) | 1 (10.0) | 2 (10.5) |

| | | | | | | | |
|---|---|---|---|---|---|---|---|
| Depression | 0 | 0 | 0 | 0 | 1 (11.1) | 0 | 1 (5.3) |
| Pruritus generalized | 0 | 0 | 1 (10.0) | 1 (3.7) | 0 | 0 | 0 |
| Pruritus allergic | 0 | 0 | 0 | 0 | 0 | 1 (10.0) | 1 (5.3) |
| Rash | 0 | 0 | 1 (10.0) | 1 (3.7) | 0 | 0 | 0 |
| Rash erythematous | 0 | 0 | 0 | 0 | 0 | 1 (10.0) | 1 (5.3) |
| Rash follicular | 0 | 0 | 1 (10.0) | 1 (3.7) | 0 | 0 | 0 |
| Urticaria | 1 (11.1) | 0 | 0 | 1 (3.7) | 0 | 0 | 0 |

[a]Safety analysis set included all patients who received ≥1 dose of study drug.

[b]Reasonable possibility that the event may have been caused by the study drug as assessed by the investigator; if relationship was missing, the event was considered drug-related. ALT, alanine aminotransferase; AST, aspartate aminotransferase; ECG, electrocardiogram; NA, not applicable.

**TABLE S4** Details of treatment-emergent adverse events of particular interest that were at least possibly related to study drug

| Patient | Isavuconazonium sulfate dose (mg) | TEAE(s) | Serious/ nonserious | Severity | Relation to study drug | Details of TEAE |
|---|---|---|---|---|---|---|
| **IV cohort** | | | | | | |
| 16-year-old girl | 372 | Prolonged QT interval | Serious (as judged by the investigator) | Moderate | Possible | A single episode of prolonged QT interval was experienced on day 7. ECG and HR results were as follows: day −3 [baseline], QTc = 479 msec, HR = 98 bpm; day 1, sinus tachycardia, unable to rule out infarct; day 7, QTc = 484 msec, HR not reported; ECG on day 8 showed sinus tachycardia (HR = 106 bpm), non-specific T-wave abnormality, and QTc shorter than baseline on day -3 (QTc = 457). The prolonged QTc was classed as moderate in severity; study drug was discontinued on day 8; treatment was not required, but concomitant famotidine, diphenhydramine and promethazine hydrochloride were discontinued as they may affect QTc |

| Patient | Isavuconazonium sulfate dose (mg) | TEAE(s) | Serious/ nonserious | Severity | Relation to study drug | Details of TEAE |
|---|---|---|---|---|---|---|
| | | | | | | values. Next ECG done on day 15 showed resolution of AE (QTc = 456 msec). |
| 6-year-old girl | 185.5 | Increased hepatic enzymes | Nonserious | Severe | Probable | ALT and AST levels were 11 U/L and 8 U/L at screening (day −3). Levels of these enzymes increased on day 11 (laboratory results not reported). ALT and AST levels were observed to be ≥10 and ≥3 times the ULN on day 14; 476 U/L and 196 U/L respectively. Study drug was discontinued on day 15, by which time both ALT and AST had improved substantially; 352 U/L and 92 U/L respectively. The event was classed as severe. The patient had received no potentially hepatotoxic concomitant medications. AP and total bilirubin levels remained low and unaffected (AP 134–181 U/L, bilirubin 0.34–0.65 mg/dL). ALT and |

| Patient | Isavuconazonium sulfate dose (mg) | TEAE(s) | Serious/ nonserious | Severity | Relation to study drug | Details of TEAE |
|---|---|---|---|---|---|---|
| | | | | | | AST were considered normalized by day 24. |
| 10-year-old boy | 336.5 | Increased hepatic enzymes | Nonserious | Mild | Probable/possible | At screening (day −5), ALT and AST were 18 U/L and 20 U/L, respectively. Levels were elevated on days 15 to ALT = 169 U/L and AST = 90 U/L; on day 29 (EOT), ALT = $10^9$ U/L and AST = 44 U/L. There were no significant changes in total bilirubin (0.21–0.77 mg/dL) or AP levels (94–134 U/L). |
| 4-year-old girl | 168 | Infusion-related reactions | Nonserious | Mild | Possible | Chills and nausea during infusion on day 1. Chills, vomiting, and diarrhea during infusion on day 3; treatment was not discontinued. |
| 3-year-old boy | 152 | Infusion-related reactions | Nonserious | Mild | Possible | Nausea, vomiting, and abdominal pain during infusion on day 1; treatment was not discontinued. |
| **Oral cohort** | | | | | | |

| Patient | Isavuconazonium sulfate dose (mg) | TEAE(s) | Serious/ nonserious | Severity | Relation to study drug | Details of TEAE |
|---|---|---|---|---|---|---|
| 10-year-old girl | 372.5 | Tachycardia, nausea, vomiting and pyrexia | Serious (required hospitalization) | Mild; severe | Possible | Tachycardia, nausea, vomiting and pyrexia (all classed as serious due to requirement for hospitalization) on day 18; pyrexia was classed as mild in severity, while tachycardia, nausea and vomiting were classed as severe. Tests for viral and bacterial infection on day 18 were negative. Nausea, vomiting and pyrexia resulted in discontinuation of study drug on day 20; a chest X-ray on day 21 showed a possible viral infection and intravenous rituximab was given once for EBV viremia. Pyrexia and tachycardia resolved by day 22; nausea and vomiting resolved by day 39. |
| 7-year-old girl | 223.5 | Increased hepatic enzymes | Nonserious | Moderate | Deemed not related by investigator, and possibly related by industrial sponsor | ALT and AST were 29 U/L and 45 U/L at screening (day 1). On day 22, ALT = 140 U/L and AST = 121 U/L; levels rose further to 181 U/L and 146 U/L, respectively, on day 23 when study drug was discontinued. |

| Patient | Isavuconazonium sulfate dose (mg) | TEAE(s) | Serious/ nonserious | Severity | Relation to study drug | Details of TEAE |
|---|---|---|---|---|---|---|
| | | | | | | The event was classed as moderate in severity. ALT and AST levels normalized by day 71. There were no significant changes in total bilirubin or AP levels (AP 99–210 U/L, bilirubin 0.20–0.40 mg/dL). |
| 10-year-old girl | 298 | Upper abdominal pain | Nonserious | Moderate | Possible | A single episode of upper abdominal pain on day 9, classified as moderate in severity. Study drug was discontinued the same day and the event resolved on day 10. |

[a]A serious AE was defined as an AE that resulted in death; was life-threatening; caused persistent or significant disability/incapacity, a congenital abnormality, or a birth defect; led to hospitalization or prolongation of hospitalization; or was another medically important event that may have jeopardized the patient or required intervention to prevent one of the other outcomes listed in the definition above. The investigator evaluated relatedness of AEs to study drug (not related, possible, or probable), the severity of AEs, and resolution of AEs. AE, adverse event; ALT, alanine aminotransferase; AP, alkaline phosphatase; AST, aspartate aminotransferase; EBV, Epstein–Barr Virus; ECG, electrocardiogram; EOT, end of treatment; HR, heart rate; IV, intravenous; QTc, corrected QT interval; TEAE, treatment-emergent adverse event.

**TABLE S5** Inclusion and exclusion criteria

**Inclusion criteria:**

- Male or female patients aged 1 year to <18 years for intravenous (IV) cohorts or 6 to <18 years for oral cohorts at risk for invasive mycoses
- Adequate venous access for drug administration (IV cohorts) and blood sampling
- For oral cohorts, patient was able to swallow the oral capsule of study medication
- Females of childbearing potential had a negative pregnancy test at screening and agreed not to become pregnant or to breastfeed during the study and for 28 days after final study drug administration
- Agreement by patients of childbearing potential who were heterosexually active to use two forms of birth control (≥1 of which had to be a barrier method) during the study and for 28 days (females) or 90 days (males) after final study drug administration
- Agreement not to donate ova or sperm during the study and for 28 days (ova) or 90 days (sperm) after final study drug administration
- Agreement not to participate in another interventional trial while on study treatment
- Written informed consent provided by the patient and/or parent(s) or legal guardian prior to any study-related procedures

**Exclusion criteria:**

- Familial short QT syndrome, receipt of medication known to shorten QT interval, or clinically significant abnormal electrocardiogram
- Hepatic dysfunction
- Use of strong CYP3A4 inhibitors or inducers within 5 days of first study drug administration
- History of allergy, hypersensitivity, or serious reaction to azole antifungal agents
- Anticipated survival <30 days
- Receipt of investigational therapy, except in oncology drug trials, within 28 days or 5 half-lives, whichever was longer, prior to screening
- For oral cohorts, gastrointestinal disease or condition expected to interfere with oral absorption or tolerance of study drug
- Previous dosing with isavuconazonium sulfate
